# A longan yield estimation approach based on UAV images and deep learning

**DOI:** 10.3389/fpls.2023.1132909

**Published:** 2023-03-06

**Authors:** Denghui Li, Xiaoxuan Sun, Yuhang Jia, Zhongwei Yao, Peiyi Lin, Yingyi Chen, Haobo Zhou, Zhengqi Zhou, Kaixuan Wu, Linlin Shi, Jun Li

**Affiliations:** ^1^ College of Engineering, South China Agricultural University, Guangzhou, China; ^2^ Guangdong Laboratory for Lingnan Modern Agriculture, Guangzhou, China; ^3^ Key Laboratory of South China Agricultural Plant Molecular Analysis and Genetic Improvement, Guangdong Provincial Key Laboratory of Applied Botany, South China Botanical Garden, Chinese Academy of Sciences, Guangzhou, China; ^4^ South China National Botanical Garden, Guangzhou, China; ^5^ University of Chinese Academy of Sciences, Beijing, China

**Keywords:** yield estimation, UAV image, convolutional neural network, image analysis, regression analysis

## Abstract

Longan yield estimation is an important practice before longan harvests. Statistical longan yield data can provide an important reference for market pricing and improving harvest efficiency and can directly determine the economic benefits of longan orchards. At present, the statistical work concerning longan yields requires high labor costs. Aiming at the task of longan yield estimation, combined with deep learning and regression analysis technology, this study proposed a method to calculate longan yield in complex natural environment. First, a UAV was used to collect video images of a longan canopy at the mature stage. Second, the CF-YD model and SF-YD model were constructed to identify Cluster_Fruits and Single_Fruits, respectively, realizing the task of automatically identifying the number of targets directly from images. Finally, according to the sample data collected from real orchards, a regression analysis was carried out on the target quantity detected by the model and the real target quantity, and estimation models were constructed for determining the Cluster_Fruits on a single longan tree and the Single_Fruits on a single Cluster_Fruit. Then, an error analysis was conducted on the data obtained from the manual counting process and the estimation model, and the average error rate regarding the number of Cluster_Fruits was 2.66%, while the average error rate regarding the number of Single_Fruits was 2.99%. The results show that the method proposed in this paper is effective at estimating longan yields and can provide guidance for improving the efficiency of longan fruit harvests.

## 1 Introduction

Smart orchard systems can effectively evaluate the growth conditions of fruit trees and improve the quality of fruits through digital technology. During the fruit ripening period, accurate statistics regarding the output of each fruit tree and the total output of the whole orchard can not only improve the efficiency of deploying harvesting robots and transportation robots but also guide market pricing and upgrade the fruit yield grade, which is conducive to the maximization of the economic benefits of orchards (He et al., 2022). Longan is widely studied in the field of smart orchards. At present, the yield estimation methods for longan orchards mainly adopt manual visual investigation. This statistical method is labor-intensive, laborious and time-consuming; is easily influenced by the subjective factors of different investigators; and has low accuracy and efficiency (Marani et al., 2021). Therefore, to reduce the cost of longan orchard yield estimation and improve the accuracy of yield estimation, it is necessary to develop a system that can automatically estimate longan orchard yields.

**Figure 1:**
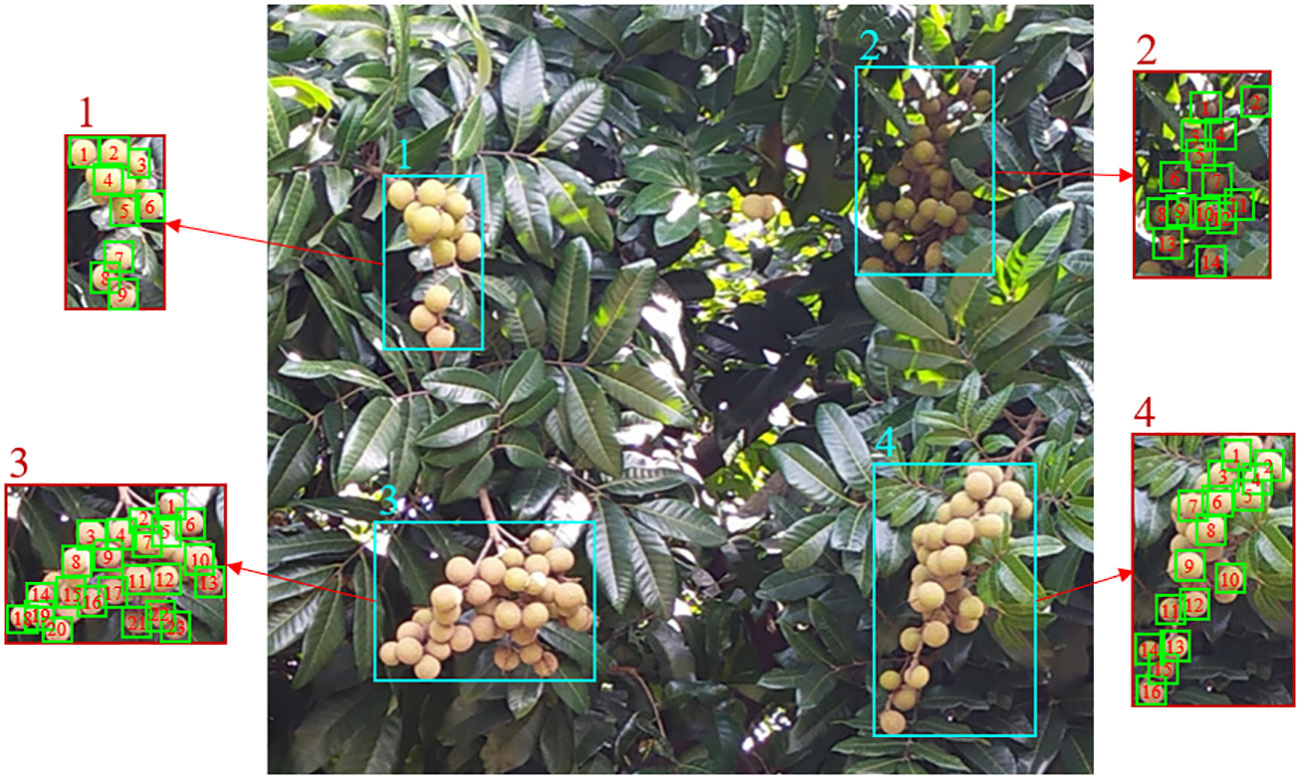
Growth characteristics of longan fruits and their quantitative statistical scheme.

Longan fruits are usually clustered and grow on the outside of the canopy (Pham et al., 2015). The growth characteristics of fruits and their quantitative statistical scheme are shown in **Figure 1**. In the natural environment, the distribution of longan fruits is complex, and they are easily blocked by leaves and branches, exhibiting different postures in different growing environments. Therefore, accurate longan fruit detection is the difficult part of realizing automatic yield estimation, which directly affects the accuracy and efficiency of orchard yield estimation. In recent years, researchers in related fields have used shape matching, color space transformation, threshold segmentation, multiscale feature fusion, fuzzy clustering and other methods to identify, detect and classify apples, oranges and other fruits (Jaisin et al., 2013; Xiong et al., 2018; Zhuang et al., 2019; He et al., 2020; Lin et al., 2020). The traditional machine learning methods used in these studies can only be used for image processing tasks with simple background conditions, and have poor robustness in the face of very complex actual orchard scenes.

With the development of sensor and computer technology, deep learning approaches have been widely developed and applied by researchers, and deep learning exhibits an excellent learning ability in cases involving the extraction of features from complex images. In recent years, with the demand for intelligence in the agricultural field, an increasing number of researchers have used deep learning technology to process collected image data for various tasks (Wang et al., 2019; da Silva et al., 2021; de Medeiros et al., 2021; Zhou et al., 2022), including fruit recognition (Gao et al., 2020; Xiong et al., 2020), classification of plants (Flores et al., 2021), classification of pests and diseases (Anagnostis et al., 2021; Singh et al., 2021), monitoring of crop growth state based on remote sensing (Ma et al., 2019; Paoletti et al., 2019), nondestructive testing and grading of fruit (Koirala et al., 2019), and animal behavior analysis (Norouzzadeh et al., 2018). To sum up, the deep learning model has stronger feature extraction ability, and it can effectively solve complex nonlinear problems. Faced with the problems of complex image backgrounds, uneven light intensities and diverse fruit features in complex orchard scenes, some researchers have applied deep learning technology to target detection tasks in complex scenes, and these models have strong robustness (Alpaydin, 2016; Liang et al., 2020; Li et al., 2021; Zhong et al., 2021; Wu et al., 2022; Tang et al., 2023).

How to quickly obtain high-definition images of orchard scenes is a key issue for improving the efficiency of orchard yield estimation. With the rapid development of unmanned aerial vehicle (UAV) power systems, control systems and sensor technology, it is possible for UAVs to carry various types of sensors to observe the earth. In recent years, UAVs have been equipped with various sensors and used in agriculture, including plant growth state detection and yield estimation (Vanegas et al., 2018; Tetila et al., 2020; Zhou et al., 2020; Feng et al., 2020a; Feng et al., 2020b; Sumesh et al., 2021). Therefore, the researchers on our team use an RGB camera on a UAV to plan the UAV flight path in advance and quickly obtain high-definition image data about the orchard.

In this study, by combining a UAV and deep learning application technology, a fast and accurate longan yield statistics approach is proposed. This approach will help to improve the accuracy and efficiency of the yield statistics of each longan tree in the modern orchard production scene and provide information for the task assignments of fruit picking UAVs and fruit transport aircraft. The main contributions of this research are as follows.

- A method of collecting canopy images and videos of each fruit tree with a UAV is proposed to accurately and completely obtain canopy image data for each fruit tree.
- Two datasets are set up to train and evaluate the performance of different target detection models.
- A scheme for counting the numbers of different targets is proposed; this scheme includes a model based on Cluster_Fruit-YOLOv5s_Deepsort (CF-YD) and a model based on Single_Fruit-YOLOv7_Deepsort (SF-YD).
- A regression analysis is carried out on the quantities counted by the two models and their real quantities to obtain a fitting equation.

The main contents of this paper are as follows: Section 2 introduces the materials and methods, Section 3 introduces the model construction process and the statistical strategy for calculating single fruit tree yields, Section 4 introduces the model experiment and results analysis in detail, and Section 5 summarizes the full text.

## 2 Materials and methods

### 2.1 Overview of the fruit tree yield estimation methods

**Figure 2:**
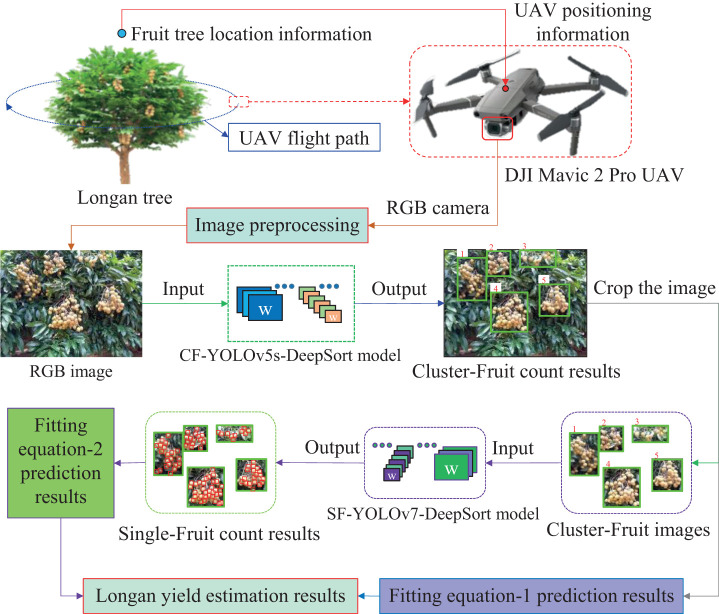
Solution for longan fruit tree yield statistics.

**Figure 2** shows the method of quickly and accurately calculate the yield of each fruit tree. First, fruit tree canopy images are collected, and the obtained images are preprocessed as the Cluster_Fruit image dataset. Second, a two-step model is established. The first step is to count the number of Cluster_Fruits on a single fruit tree based on the CF-YD model, and the second step is to count the number of Single_Fruits on each Cluster_Fruit based on the SF-YD model. The results of each step are combined with the corresponding fitting equation to correct the final result.

In the two-step model of this scheme, when each Cluster_Fruit is detected from a canopy image of fruit trees based on the CF-YD model, each Cluster_Fruit needs to be cut out as the input image of the statistical Single_Fruit quantity model. After using the SF-YD model to count the number of Single_Fruits in each image cut out in the previous step, it is necessary to calculate the yield of a single fruit tree according to the weight of the Single_Fruit.

### 2.2 Sensor system and image acquisition

**Figure 3:**
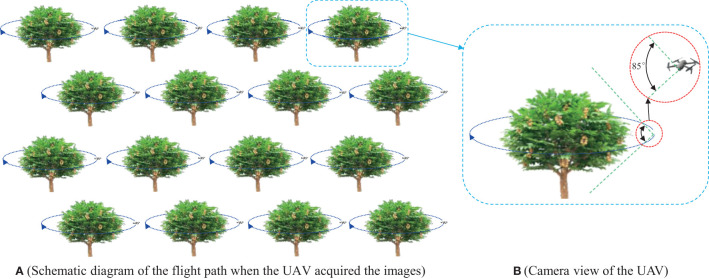
Schematic diagram of the image acquisition mode.

To build the data set, 1100 valid longan images and 16 complete longan tree canopy videos were acquired at the Longan orchard in Guangzhou on July 1-25, 2021, and July 1-26, 2022, during two different time periods: morning (7:30–11:30) and afternoon (13:30–18:30). Furthermore, to perform modelling and verify the accuracy of the model, the actual numbers of Cluster_Fruits and Single_Fruits and the quality of 16 Cluster_Fruits from 16 fruit trees in the orchard were manually counted. A lightweight, small-size and high-resolution RGB camera mounted on a DJI Mavic 2 Pro UAV was used to collect orchard canopy images. The camera had 12 million pixels, the viewing angle was 85 degrees, the focal point ranged from 0.5 m to infinity, and 120 images could be taken in one second at the fastest speed. **Figure 3** is a schematic diagram of the image acquisition mode.

**Figure 4:**
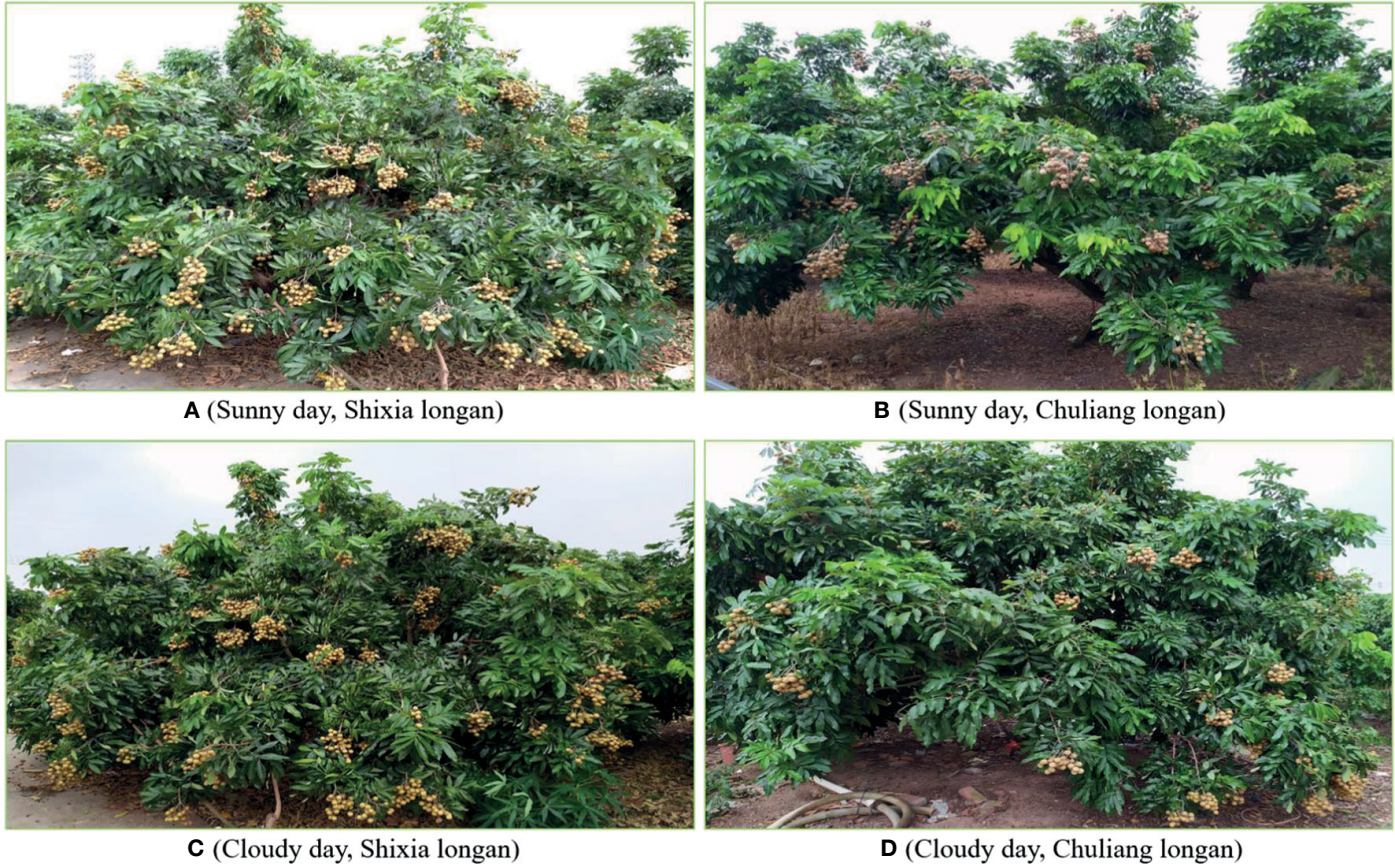
Examples of the UAV images.

According to the planting mode of modern orchards, the images were collected according to the following steps. ① Control the UAV to fly around the fruit trees, and during this process, have the RGB camera mounted on the UAV always look straight at the tree center to collect the canopy images of the fruit trees. ② Set the RGB image resolution to 1280×720 pixels, and automatically save each image to the image acquisition card obtaining it. In order to ensure the diversity of images in the data set, images were taken on sunny and cloudy days respectively, including images of Shixia longan and Chuliang longan. **Figure 4** shows the examples of the UAV images.

### 2.3 Image preprocessing

**Figure 5:**
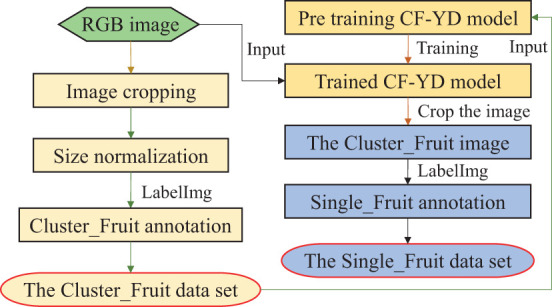
Diagram of the whole process.

In this study, data sets are prepared for the two-step model. First, aiming at the images collected by the camera on the UAV, image cropping and size normalization are performed, and the bounding box of each Cluster_Fruit in each sample is manually marked, forming a Cluster_Fruit data set for the CF-YD model. Second, the trained CF-YD model is used to detect Cluster_Fruits in the original RGB images, each Cluster_Fruit image is cut out according to the output coordinate information, and the Single_Fruit positions in each image are manually marked to form a Single_Fruit data set for the SF-YD model. **Figure 5** shows the whole process.

#### 2.3.1 Construction of the Cluster_Fruit image data set

During the process of constructing the Cluster_Fruit image dataset, firstly, to ensure the diversity of the training samples, a self-programmed random cropping algorithm is used to amplify the Cluster_Fruit images. After the initial longan dataset is expanded, 1100 longan images are obtained. Then, the image size is normalized to 1280×1280 pixels. Finally, the 1100 images are manually annotated following the guidelines of the Pascal VOC 2010 reference challenge. The labelling information mainly includes the size of each image, the target category and the specific position coordinate information of the target area.

#### 2.3.2 Construction of the Single_Fruit image dataset

As the SF-YD model adopted in this study first normalizes the input images of any size to 640×640 pixels, the trained CF-YD model is used to detect each image in the Cluster_Fruit data set, and the coordinate information of each Cluster_Fruit image is obtained and directly cut out from the original image. After screening, 1100 Cluster_Fruit images are selected. LableImg software is used to manually mark the positions of Single_Fruits in each image to form a Single_Fruit data set for training and testing the SF-YD model.

In summary, the Cluster_Fruit data set and Single_Fruit data set are constructed by the above two methods. The images in both data sets are divided into a training set, verification set, and test set according to a ratio of 8:1:1. **Table 1** presents the images and the annotation information.

**Table 1:** Details of the Cluster_Fruit and Single_Fruit data sets.

Data set	Cluster_Fruit data set		Single_Fruit data set	
	Images	Cluster_Fruit	Images	Single_Fruit
Full data set	1100	11239	1100	24045
Training data set	880	8898	880	19393
Validation data set	110	1205	110	2285
Test data set	110	1136	110	2367

## 3 Model construction and statistical strategy for fruit tree yield estimation

In this section, according to the video image data of the canopy of a single fruit tree collected by the RGB camera on the UAV, a yield prediction scheme for a single fruit tree is proposed. First, according to the growth characteristics of Cluster_Fruit, the improved YOLOv5s target detection algorithm and Deepsort target tracking algorithm are incorporated into the CF-YD model, and the flow chart for quickly and accurately obtaining the numerical and location information of Cluster_Fruits from the video image of a single fruit tree is determined. Then, according to the growth characteristics of Single_Fruits, the YOLOv7 target detection algorithm and Deepsort target tracking algorithm are merged into the SF-YD model, and the flow chart for quickly obtaining the number of Single_Fruits from a video image of Cluster_Fruit is worked out. Finally, according to the prediction results regarding the quantities of Cluster_Fruits and Single_Fruits, a strategy for counting the output of a single fruit tree is proposed.

### 3.1 Deepsort algorithm

The Deepsort algorithm (Wojke et al., 2017) is an algorithm with a multitarget tracking function, which is improved on the basis of the SORT algorithm (Bewley et al., 2016). Compared with the SORT algorithm, the Deepsort algorithm improves the content matching process to avoid ignoring multitarget ID transformations, uses appearance information to curb the frequency of target ID transformations, and adds a simple convolutional neural network (CNN) model to extract the appearance features of detected targets (expressed by low-dimensional vectors). The core of the Deepsort algorithm consists of prediction, observation and updating. The specific flow of the Deeppart algorithm is as follows. ① The target information predicted by You Only Look Once (YOLO) is input into the Deeppart algorithm as the observed value. The Kalman filter first judges whether a track is present, and if one is, it predicts the prior probability of the target information, then carries out cascade matching and IoU matching in the matching module, and finally obtains the matching success list. ② In the Kalman updating module, a posteriori prediction is performed on the successfully matched target to obtain the corrected target coordinates, and parameters such as the Kalman gain are updated. ③ The above operations are repeated until all the videos are processed.

### 3.2 CF-YD model for Cluster_Fruit statistics

The clusters of longan fruits are usually distributed in the canopies of fruit trees in disordered arrangements, and their sizes become very different as the distance from the camera increases. When quickly and accurately counting the number and locations of longan Cluster_Fruits from the canopy images of fruit trees, it is necessary to overcome the problem that target scale changes greatly affect the resulting detection accuracy. The most commonly used target detection algorithms are the R-CNN series (Girshick et al., 2015a; Girshick, 2015b; Ren et al., 2017) and YOLO series (Redmon et al., 2016; Redmon & Farhadi, 2018; Bochkovskiy et al., 2020) models. R-CNN series algorithms, also known as target detection algorithms based on candidate areas, first generate candidate areas that may contain objects and then further classify and calibrate the candidate areas to obtain the final detection results. During the training process, YOLO series algorithms can pay more attention to the global information and the whole image in target detection. The core idea of YOLO is to use the whole picture as the input of the network and directly return to the position and category of the bounding box at the output.

Compared with the classic YOLOv3 algorithm, the data enhancement step of the YOLOv5 detection algorithm uses Mosaic to expand the input dataset, and it can also perform operations such as flipping, brightness adjustment and clipping. For a sample set with less data, the data can be effectively expanded. Four versions of YOLOv5 are available, namely, YOLOv5s, YOLOv5m, YOLOv5l and YOLOv5x, among which YOLOv5s is the network with the smallest depth and the smallest characteristic map width in this series of detection networks.

YOLOv5s mainly consists of three parts: a backbone, a neck and an output. The backbone is the basic feature extraction layer, which is used to extract feature information from images. It includes four modules: Focus, CBH, CSP1-x and spatial pyramid pooling (SPP) modules (He et al., 2015). The neck is a feature fusion layer whose function is to fuse image information with different scales to obtain better detection results. It uses the rectified linear unit (ReLU) activation function and adopts a feature pyramid network (FPN) (Lin et al., 2017) + PAN (Liu et al., 2018) network structure. The output is the output layer, whose function is to output the predicted target information, in which nonmaximum suppression (NMS) is performed on the last detection frame of the target to obtain the optimal target frame; three different detection scales (20×20, 40×40, 80×80) are provided, which can predict longan Cluster_Fruits with different sizes. In the early stage, our team improves the YOLOv5s model for the Cluster_Fruit detection task and improves the accuracy of the model in the target detection task.

**Figure 6:**
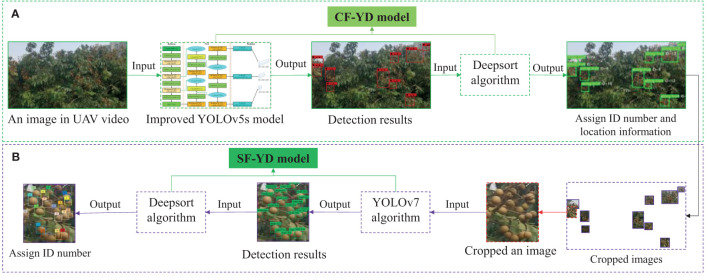
Flow chart of Cluster_Fruits and Single_Fruits quantity prediction. **(A)** The flow chart of Single_Fruit quantity statistics in a Cluster_Fruit, **(B)** The flow chart of Cluster_Fruit quantity statistics in a single fruit tree.

In this paper, according to the growth characteristics of Cluster_Fruit, the improved YOLOv5s target detection algorithm (Li et al., 2022) and Deepsort target tracking algorithm are incorporated into the CF-YD model. **Figure 6A** shows the flow chart for the numerical Cluster_Fruit statistics in a single fruit tree. First, after the complete video data of a single fruit tree are input into the YOLOv5s algorithm, the YOLOv5s algorithm detects multiple targets in each frame and inputs the position information obtained from multiple Cluster_Fruits into the Deepsort algorithm to assign ID numbers. Then, the correlation filtering algorithm is used to compare whether anchor frames with the same size are present in the front and back frames (a target with the same anchor frame size continues to use the original number and assigns a new ID number to the new target). Finally, the maximum ID number is output, and the value of this number is used as the number of predicted Cluster_Fruits in the fruit tree canopy.

### 3.3 SF-YD model for Single_Fruit statistics

The Single_Fruits of longan are usually distributed in Cluster_Fruits in a disorder manner and occupy very small areas in the canopy images of fruit trees, so it is difficult to accurately count the number of Single_Fruits in each Cluster_Fruit directly from the canopies of fruit trees. In this study, the latest YOLOv7 algorithm (Wang et al., 2022), which has a higher detection accuracy and a faster detection speed than other algorithms in the series, is adopted to realize the Single_Fruit detection task. The YOLOv7 algorithm adopts strategies such as the extended efficient long-range attention network (E-ELAN), model scaling based on concatenation-based models (Wang et al., 2021), and convolutional reparameterization (Ding et al., 2021) and achieves a very good balance between detection efficiency and accuracy.

The YOLOv7 network mainly includes four parts: an input, a backbone, a head and a prediction module. The input module normalizes the input image of any size to the input pixel size set by the backbone network. The backbone module consists of several BConv layers, E-ELAN layers and MPConv layers. The BConv layer consists of a convolution layer, a batch normalization layer and a LeakyReLU activation function (Jiang & Cheng, 2019), which is used to extract image features with different scales. The E-ELAN layer keeps the original ELAN design framework and improves the learning ability of the network without destroying the original gradient path by guiding the computing blocks of different feature groups to learn more diverse features. On the basis of the BConv layer, the MPConv layer adds an Maxpool layer to form two branches. The upper branch cuts the image length and width by half through Maxpool and the image channel by half through the BConv layer. In the lower branch, the image channel is halved by the first BConv layer, and the image length and width are halved by the second BConv layer. Finally, the features extracted by the upper and lower branches are fused by the Cat operation, which improves the feature extraction ability of the network. The head module is composed of a path aggregation FPN (PAFPN) (Ge et al., 2021) structure. By introducing the bottom-up path, the bottom-up information can be transmitted to the higher level more easily, thus realizing the efficient integration of features at different levels. The prediction module adjusts the number of image channels for three features of the PAFPN output with different scale, such as P3, P4 and P5, through the REPVGG block structure and finally uses a 1×1 convolution to predict the confidence, category and anchor frame.

According to the growth characteristics of Single_Fruits, the YOLOv7 target detection algorithm and Deepsort target tracking algorithm are incorporated into the SF-YD model. **Figure 6B** shows the flow chart of the numerical Single_Fruit statistics in a Cluster_Fruit. First, each Cluster_Fruit with an ID number assigned by the CF-YD model is continuously cut out from the original video image to form Cluster_Fruit video data with ID numbers. The YOLOv7 algorithm detects multiple targets in each frame, inputs the obtained position information of multiple Single_Fruits into the Deepsort algorithm to assign ID numbers, then compares whether anchor frames of the same size are present in the previous and subsequent frames by using the correlation filtering algorithm (targets with the same anchor frame size continue to use the original number, and new ID numbers are assigned to new targets), and finally outputs the maximum ID number and takes the value of this number as the number of Single_Fruits in each Cluster_Fruit.

### 3.4 Statistical strategy for a single fruit tree yield

In this section, according to the prediction results regarding the numbers of Cluster_Fruits and Single_Fruits in the previous two sections, the specific steps of longan yield estimation are formulated:

(1) Step 1: The CF-YD model is used to quickly obtain the number *N_CF_*_1_ of Cluster_Fruits from the canopy images of fruit trees, and the total number *N_CF_*_2_ of Cluster_Fruits is predicted by establishing a regression analysis model with the number of Cluster_Fruits counted in the real orchard.
(2) Step 2: The location information of the *N_CF_*_1_ Cluster_Fruits obtained in the previous step is cut from the original image in turn and input into the SF-YD model to obtain the total number *N_SF_*_1_ of Single_Fruits. The total number *N_SF_*_2_ of Single_Fruits is predicted by establishing a regression analysis model with the number of Single_Fruits in the *N_CF_*_1_ Cluster_Fruit statistics in the actual orchard.
(3) Step 3: According to the total number *N_SF_*_2_ of Single_Fruits in *N_CF_*_1_ Cluster_Fruits, the average number *AVE_nSF_* of Single_Fruits in a single cluster can be calculated. Ten Cluster_Fruits are randomly selected to weigh and count the number of Single_Fruits and calculate the average quality *AVE_mSF_* of Single_Fruits.
(4) Step 4: The formula for calculating the yield TQ of a single fruit tree is:

(1) $$\text{TQ}=\;(N_{SF2}+\;\left(N_{CF2}-N_{CF1}\right)*AVE_{nSF})*\;AVE_{mSF}$$

## 4 Model experiment and results analysis

### 4.1 Model training and parameter design

The training and testing processes of the CF-YD and SF-YD models are implemented on a workstation with the Ubuntu 18.04 LTS operating system. The main hardware devices of the workstation are as follows: GPU: NVIDIA GTX3060 (configured with CUDA 10.1 and cuDNN 7.1); processor: 11^th^ Gen Intel (R) Core (TM) i7-11800H; RAM: NVIDIA 16G; and hard disk: Samsung 1T. On the PyTorch deep learning framework, a CNN model is built with the Python programming language.

The image size of the CF-YD model training data is set to 1280×1280 pixels. In terms of parameter settings, the intersection over union (IoU) is set to 0.5, the initial learning rate is 1e-4, and the learning rate at the end of training is set to 1e-5. The dataset is split at a 90-10 training-verification ratio, and a total of 500 epochs of iterative training are conducted. During the training process, after conducting a series of convolution and pooling operations with the CF-YD model, the input image uses the anchor box in the feature map layer to extract a series of features. In the feature layer, each cell is mapped to the original image, the premarked anchor box is found, and then the loss value between this anchor box and the ground truth is calculated. After training, the CF-YD model obtains a series of model parameters to fit the real border with the anchor box.

During the training process of the SF-YD model, input images of any size are normalized to 640×640 pixels through the input module. The training process of the model is divided into the Freeze phase and UnFreeze phase, the optimizer is set to stochastic gradient descent (SGD), the initial learning rate is 0.01, the momentum is 0.9, and the weight decay is set to 5e-4. The cosine annealing algorithm is used to adjust the learning rate, and the minimum learning rate is 1e-4. The training and verification steps alternate. During the Freeze phase, the training duration is 50 epochs, each epoch has 220 iterations in the training phase and 28 iterations in the verification phase, and the batch size is 4. During the UnFreeze phase, the training duration is 250 epochs, with 440 iterations in the training phase and 55 iterations in the verification phase of each epoch, and the batch size is 2. After training, the SF-YD model obtains a series of model parameters to fit the real border with the anchor box.

**Figure 7:**
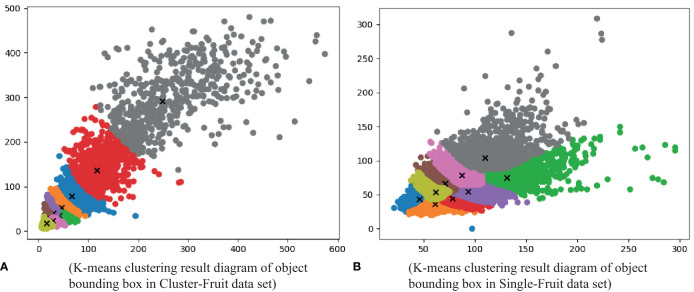
Clustering results of the target bounding boxes in the two datasets.

During the training and testing processes of the CF-YD and SF-YD models, it is necessary to generate a series of anchor boxes (candidate areas) in the given image according to certain rules. In this study, k-means clustering and a genetic algorithm are used to obtain anchor boxes. Because the prediction layer of the YOLO network contains three scales of information (corresponding to three receptive fields), each scale contains three anchors. Therefore, the YOLO network needs nine anchor scales; that is, the sizes of all the target bounding boxes in the dataset are clustered into nine categories. Through the analysis of the Cluster_Fruit and Single_Fruit datasets, the k-means clustering results of all the target bounding boxes in the two datasets are obtained. Each point in **Figure 7A** corresponds to a target bounding box in the Cluster_Fruit dataset. According to the overall size characteristics of the target bounding boxes, nine types of anchor boxes that are suitable for training and testing the CF-YD model are determined as [16,16, 21,28, 28,23, 30,39, 41,33, 46,52, 67,77, 116,135, 247,291]. Each point in **Figure 7B** corresponds to a target bounding box in the Single_Fruit dataset. According to the overall size characteristics of the target bounding box, nine types of anchor boxes that are suitable for training and testing the SF-YD model are determined as [45,43, 61,36, 61,53, 78,44, 70,66, 93,54, 87,78, 131,74, 110,104]. At the same time, it can be seen from the figure that the more points there are with the same color, the more targets with this cluster size, and the points with different colors represent targets with different cluster sizes. In other words, this figure can reflect the complicated situation regarding the targets to be detected in an orchard scene to some extent.

### 4.2 Model evaluation indicators

#### 4.2.1 Evaluation indices for the object detection algorithm

In this study, P, R, F1 score, AP, and FPS were used to evaluate the performance of two target detection models. The calculation methods for calculating the P, R, F1 score and AP here are shown in formulas (2), (3), (4) and (5).


(2) $$\text{P}=\frac{TP}{TP+FP}$$

(3) $$\text{R}=\frac{TP}{TP+FN}$$

(4) $$\text{F}1\text{ score}=\frac{2*P*R}{P+R}$$

(5) $$\text{AP}=\int_{0}^{1}P\;\left(R\right)d_{R}$$

In these formulas, TP represents true cases, FP represents false-positive cases, TN represents true-negative cases, and FN represents false-negative cases.

#### 4.2.2 Evaluation indices for the Deepsort algorithm

This study selects identity switches (IDSs), multiple-object tracking accuracy (MOTA) and multiple-object tracking precision (MOTP) to evaluate the effectiveness of the multitarget tracking algorithm. IDS is the number of times the tracking target ID changes. The smaller its value is, the better the tracking stability. MOTA considers false alarms and IDSs simultaneously and measures the performance of the tracking algorithm in terms of detecting targets and keeping track of them, which has nothing to do with target detection accuracy. The larger its value is, the better the performance of the algorithm. MOTP is used to quantify the positioning accuracy of the detector. The larger its value is, the higher the accuracy of the detector.

### 4.3 Results and discussion of the object detection and counting tasks

#### 4.3.1 Performance evaluation results of different models

To fully evaluate the performance of the CF-YD model in detecting Cluster_Fruits and the SF-YD model in detecting Single_Fruits, first, the CF-YD and SF-YD models are trained according to the training parameters set in Section 4.1, and the weight file with the best training effect in each model is used as the weight file for testing the model performance. Then, the CF-YD model for the Cluster_Fruit test set and the SF-YD model for the Single_Fruit test set comprehensively evaluated from the aspects of P, R, AP, FPS, F1 score, etc., and the obtained results are shown in **Table 2**.

**Table 2:** Evaluation index results obtained on the test dataset under different models.

Models/Evaluation index	P (%)	R (%)	AP (%)	F1 score	FPS
CF-YD model (Cluster_Fruit test data set)	83.50	85.70	82.40	0.85	56.21
SF-YD model (Single_Fruit test data set)	93.23	91.97	97.12	0.93	98.35

**Figure 8:**
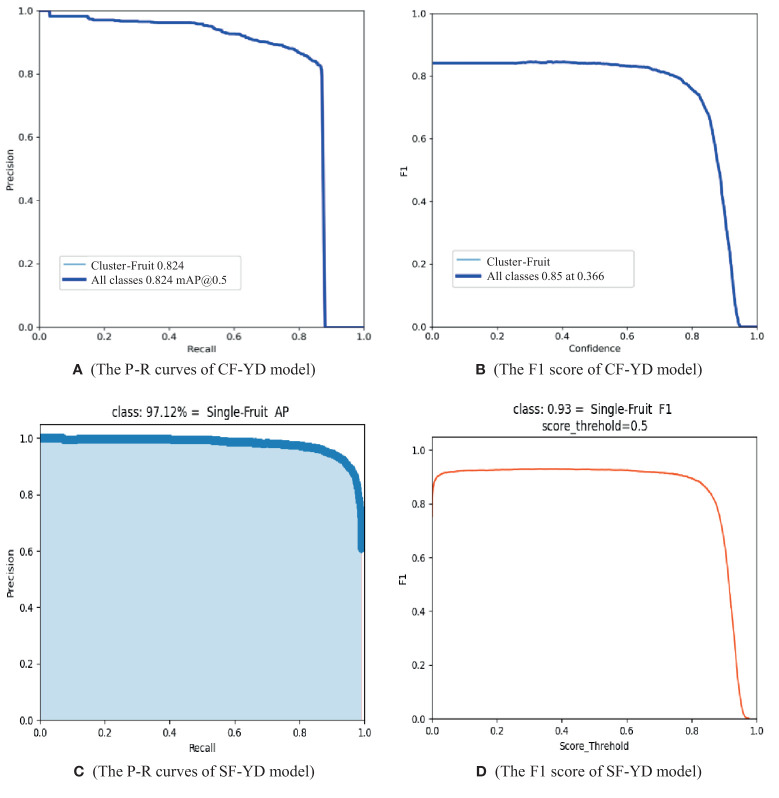
P-R curves and F1 scores of different detection methods.

The P-R curve and F1 score changes exhibited by the CF-YD model on the Cluster_Fruit test dataset are shown in **Figures 8A, B**, respectively. The area enclosed by the P-R curve and the two coordinate axes in **Figure 8A** corresponds to the AP value of Cluster_Fruit detection. As shown in **Table 2**, the AP value of the CF-YD model for Cluster_Fruit detection is 82.4% on the test set, and the detection accuracy is high. However, some Cluster_Fruits are still blocked by other Cluster_Fruits or branches and leaves and cannot be accurately detected. The F1 score in **Figure 8B** first changes slightly with increasing confidence value and then suddenly decreases sharply when the confidence value is greater than 0.7. Therefore, it is usually sufficient to set this parameter to 0.5 in the model training stage. The FPS value is the number of images that the model can detect per second, and the detection time of each image is only 18 ms. According to the clustering results of the target sizes in the Cluster_Fruit dataset, the Cluster_Fruit size exhibits the diversity characteristic. The above results show that the CF-YD model has good detection performance for multiscale targets.

The changes in the P-R curve and F1 score of the SF-YD model on the Single_Fruit test dataset are shown in **Figures 8C, D**, respectively. The area enclosed by the P-R curve and the two coordinate axes in **Figure 8C** basically covers the whole coordinate system. As shown in **Table 2**, the AP value of the SF-YD model on the test set for Single_Fruit is 97.12%, demonstrating high detection accuracy, and only a few Single_Fruits are undetected. The F1 score changes slightly with increasing confidence and suddenly decreases sharply when the confidence is greater than 0.85. Therefore, it is usually sufficient to set this parameter to 0.5 in the model training stage. The FPS value is the number of images that the model can detect per second, and the detection time of each image is only 10 ms. According to the clustering results of the target sizes in the Single_Fruit dataset, the sizes of Single_Fruits are generally small. The above results show that the SF-YD model also has good detection performance for small targets.

#### 4.3.2 Detection effects of different models in real scenes

Many varieties of longan are available, and new varieties have appeared in recent years. To further evaluate the performance of the CF-YD and SF-YD models in detecting longan Cluster_Fruits and Single_Fruits in real and complicated mountain orchard environments, this section selects images of longan orchards with different varieties (Chuliang longan and Shixia longan), different illumination conditions (Sunny day and Cloudy day), different scales and different densities, tests the trained CF-YD and SF-YD models, and obtains the detection results of each model.

**Figure 9:**
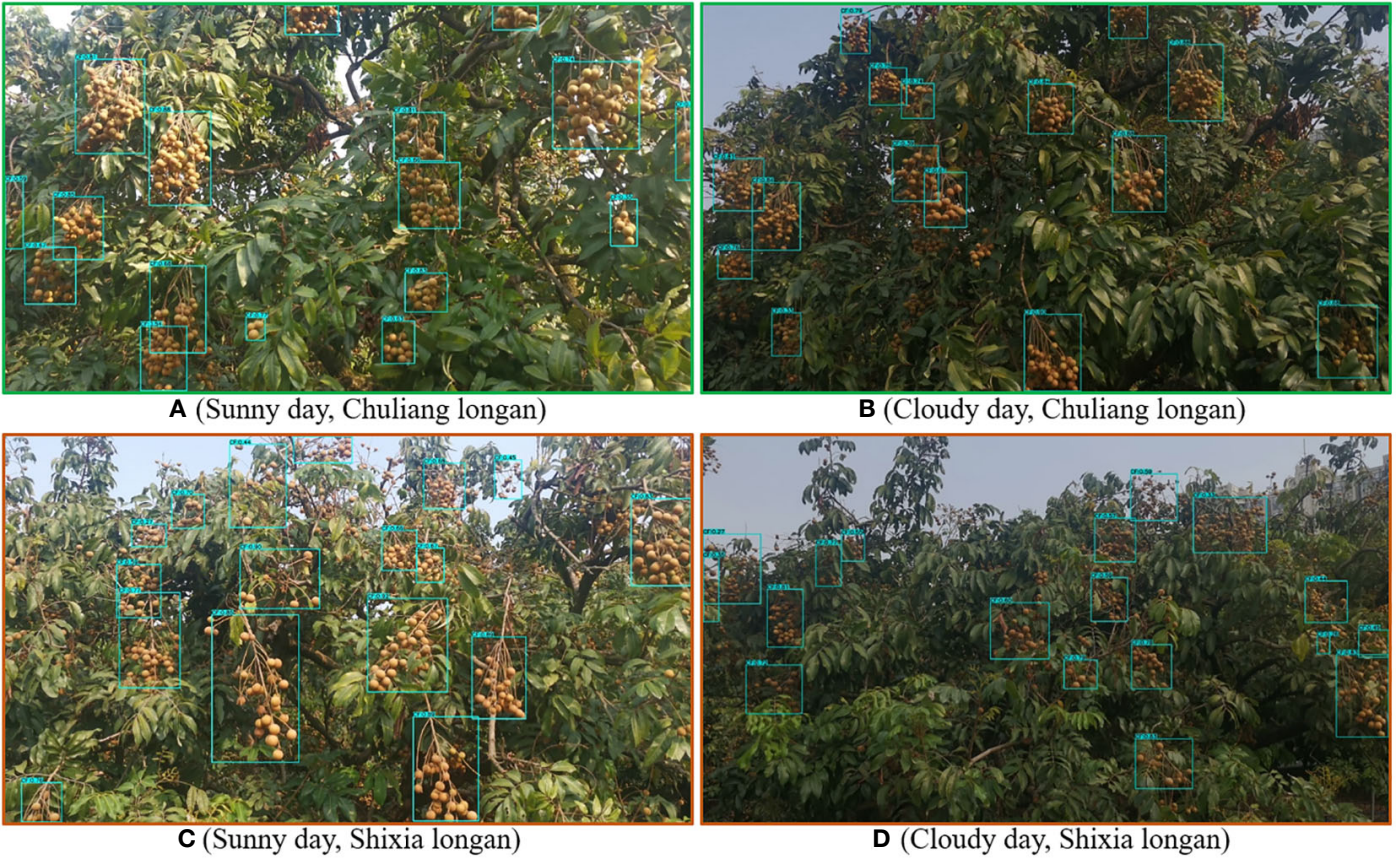
Cluster_Fruit detection results of the CF-YD model under different scenes.

**Figures 9A, B** are the test results obtained by the CF-YD model for Chuliang longan in different scenes of real orchards. **Figures 9C, D** are the test results obtained by the CF-YD model for Shixia longan in different scenes of real orchards. From the detection results, it can be seen that regardless of the longan variety and in sunny day or cloudy day, Cluster_Fruit is accurately detected for large-scale or small-scale targets. The above detection results show that the CF-YD model has good feature extraction performance, has strong generalization to different varieties of longan in real orchard environments and is not easily disturbed by uneven light. It also has a good detection effect on small targets, so it is suitable for target detection in longan orchards.

**Figure 10:**
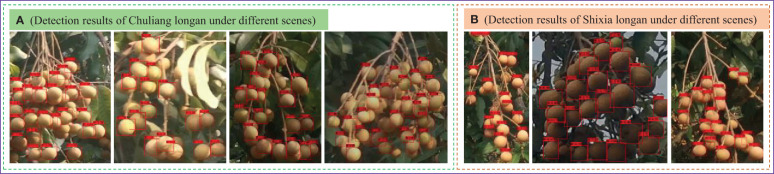
Single_Fruit detection results of the SF-YD model under different scenes.

To evaluate the detection effect of the SF-YD model on different longan varieties in a real orchard scene, the CF-YD model is first used to identify Cluster_Fruit in a real orchard scene and cut out the video data to form Single_Fruits, which are then input into the SF-YD model for Single_Fruit detection. **Figures 10A, B** are the test results obtained by the SF-YD model for Chuliang longan and Shixia longan, respectively, in different scenes of real orchards. The fruit colors and shapes of the two longan species are quite different. They exhibit different glosses at different distances and under different light. It can be seen from the detection results that Single_Fruits of different varieties are accurately detected in, different weather conditions, with different scales and in scenes with different densities. The above detection results show that the SF-YD model has good feature extraction performance, strong generalization for different varieties of longan Single_Fruits in a real orchard environment, and a good detection effect for multiscale targets, so it is suitable for small target detection in longan orchards.

#### 4.3.3 Counting results of different models in real scenes

To evaluate the tracking performance of the CF-YD model on Cluster_Fruit and the SF-YD model on Single_Fruit, a video image of a fruit tree canopy is randomly selected to test the CF-YD model, and a video image of a Cluster_Fruit is selected to test the SF-YD model. The models are comprehensively evaluated in terms of the IDS, MOTA, MOTP and other metrics, and the results obtained are shown in **Table 3**.

**Table 3:** Evaluation index results obtained by different models.

Models/Evaluation index	IDS	MOTA (%)	MOTP (%)
CF-YD model (Cluster_Fruit)	5	95.30	92.60
SF-YD model (Single_Fruit)	2	97.20	94.70

Regarding the IDS metric, the numbers of target ID changes observed during the process of tracking the target in the video images with the two models are very small at 5 and 2, respectively. The MOTA and MOTP values of the two models are basically above 90%, which shows that both tracking algorithms can track targets stably and accurately.

To further verify the performance of the CF-YD and SF-YD models in counting the numbers of longan Cluster_Fruits and Single_Fruits in the real and complicated mountain orchard environment, this section selects images of longan orchards with different varieties (Chuliang longan and Shixia longan) and different lighting scenes, tests the trained CF-YD and SF-YD models, and obtains the counting results of each model.

**Figure 11:**
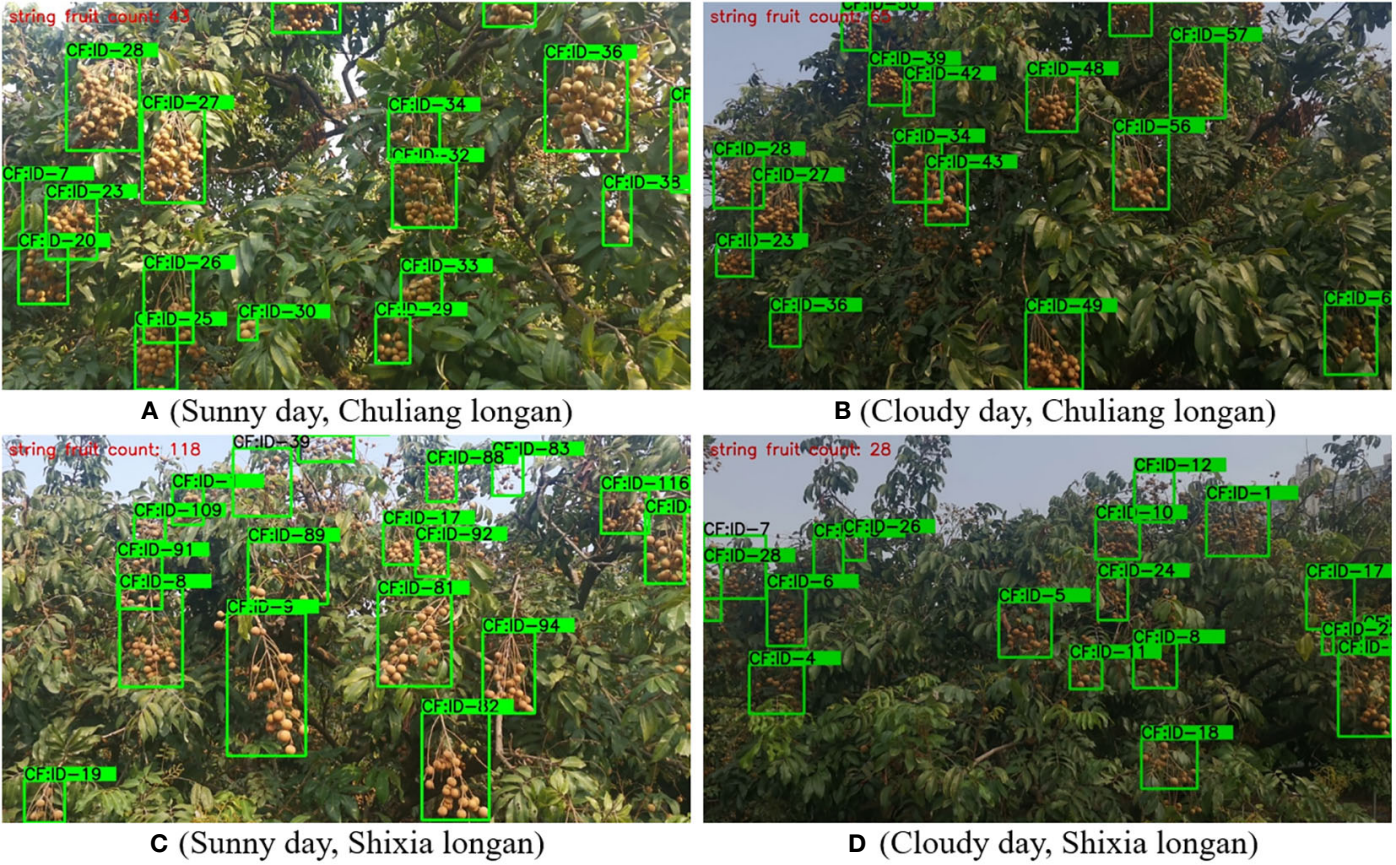
Cluster_Fruit counting results of the CF-YD model under different scenes.

**Figures 11A, B** are the Cluster_Fruit counting results obtained in different scenes of real orchards by the CF-YD model for Chuliang longan. **Figures 11C, D** show the counting results obtained by the CF-YD model for Shixia longan in different scenes of real orchards. It can be seen from the counting results that regardless of the variety and in sunny or cloudy weather, Cluster_Fruits yield accurate counting results for large-scale or small-scale targets. The above results show that the CF-YD model has good target tracking performance. It has strong generalization for different varieties of longan in real orchard environments and is not easily disturbed by uneven lighting. It also has a good tracking effect for multiple targets, so it is suitable for target counting tasks in longan orchards.

**Figure 12:**
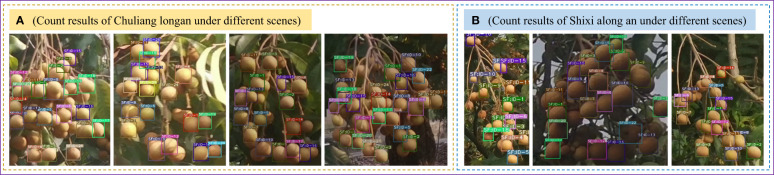
Single_Fruit counting results of the SF-YD model under different scenes.

To verify that the SF-YD model can count different longan varieties in real orchard scenes, the Single_Fruit video data are input into the SF-YD model to count Single_Fruits. **Figures 12A, B** are the counting results of the SF-YD model for Chuliang longan and Shixia longan, respectively, in different scenes of real orchards. It can be seen from the counting results diagram that the different varieties of Single_Fruits are accurately counted in different weather conditions. The above detection results show that the SF-YD model has a good target tracking performance, strong generalization for different varieties of longan Single_Fruit in a real orchard environment, and a good tracking effect for multiscale targets, so it is suitable for counting small targets in longan orchards.

### 4.4 The models for estimating the numbers of Cluster_Fruits and Single_Fruits

To accurately obtain the yield of a single longan tree, it is necessary to modify the numbers of Cluster_Fruits and Single_Fruits identified by the two models. First, 10 longan trees of different ages are randomly selected from the longan orchard, and the true number of Cluster_Fruits on each longan tree and the true numbers of Single_Fruits on the randomly selected 10 Cluster_Fruits fruits are manually counted. Then, the canopy video images of these 10 longan trees are captured by UAVs, and the number of Cluster_Fruits on each longan tree and the numbers of Single_Fruits on the 10 randomly selected Cluster_Fruits are identified by the method described in the previous section. Finally, the number of artificial statistics and the number identified by the model are fitted by an equation, and a number estimation model for the Cluster_Fruits on a single longan tree and a number estimation model for the Single_Fruits on a single Cluster_Fruit are constructed.

**Figure 13:**
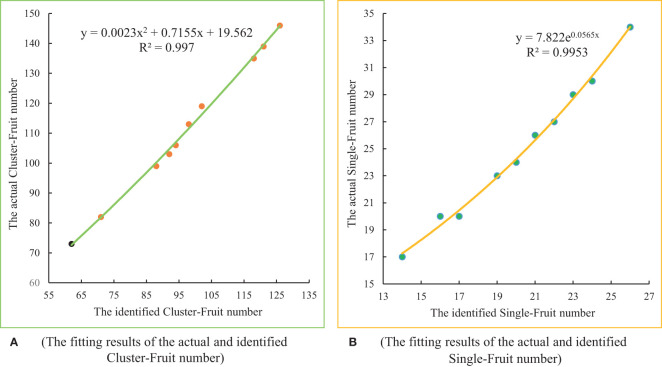
The fitting results of the actual and identified numbers of fruits.

**Table 4** counts the quantity information of the manual counting approach and two identification models. The actual value of Cluster_Fruits on 10 longan trees ranges from 91 to 312, and the actual value of Single_Fruits on ten Cluster_Fruits ranges from 18 to 32. Because the 10 longan trees and 10 Cluster_Fruits are randomly selected, the numbers of fruits will be different in different runs. At the same time, during the process of growth, the fruit of longan trees is affected by external conditions such as nutritional components and light conditions, so the yield of each tree is different. Exponential fitting, linear fitting, logarithmic fitting, binomial fitting, power fitting, etc., are performed for determining the numbers identified by the models and the actual number of manual statistics in **Table 4**. After performing a comprehensive analysis and comparing the fitting results, as shown in **Figure 13**, the best fitting method for the number of Cluster_Fruits on a single fruit tree is binomial fitting. The fitting equation is *y* = 0.0023*x* ^2^+0.7155*x*+19.562 , and the determination coefficient *R* ^2^ is 0.9970. The best fitting method for the number of Single_Fruits on a single cluster is exponential fitting, the fitting equation is *y* = 7.822*e* ^0.0565*x*^ , and the determination coefficient *R* ^2^ is 0.9953. Strong correlation is observed between the two samples.

**Table 4:** Sample number information of the two identification models and manual statistics.

Class	Identified value (Cluster_Fruit)	Actual value (Cluster_Fruit)	Class	Identified value (Single_Fruit)	Actual value (Single_Fruit)
1	275	299	1	26	34
2	283	312	2	21	26
3	261	279	3	19	23
4	213	241	4	23	29
5	195	212	5	20	24
6	153	179	6	24	30
7	182	198	7	22	27
8	93	116	8	17	20
9	113	129	9	16	20
10	77	91	10	14	17

### 4.5 Experimental results of Cluster_Fruits and Single_Fruits in real orchard scenes

To further verify the quantity estimation model in Section 4.4, six other longan trees are randomly selected from real orchards, and the true number of Cluster_Fruits on each longan tree and the true numbers of Single_Fruits on the 6 randomly selected Cluster_Fruits are obtained by manual counting. Then, the CF-YD and SF-YD models are used to obtain the identification numbers of the Cluster_Fruits and Single_Fruits from the video data, respectively. By using the fitting equation obtained in Section 4.4, the identified numbers are corrected, and the predicted numbers of Cluster_Fruits and Single_Fruits are obtained. Finally, the error between the real quantity and the predicted quantity is analyzed. The error in this study is the absolute value of the predicted value minus the actual value, and the error rate is equal to the percentage value obtained by dividing this error by the actual value. The calculation formula for the error rate is:


(6) $$\text{Error rate}=\left|\frac{\text{Predicted value}-\text{Actual value}}{\text{Actual value}}\right|\times 100\%$$

**Figure 14:**
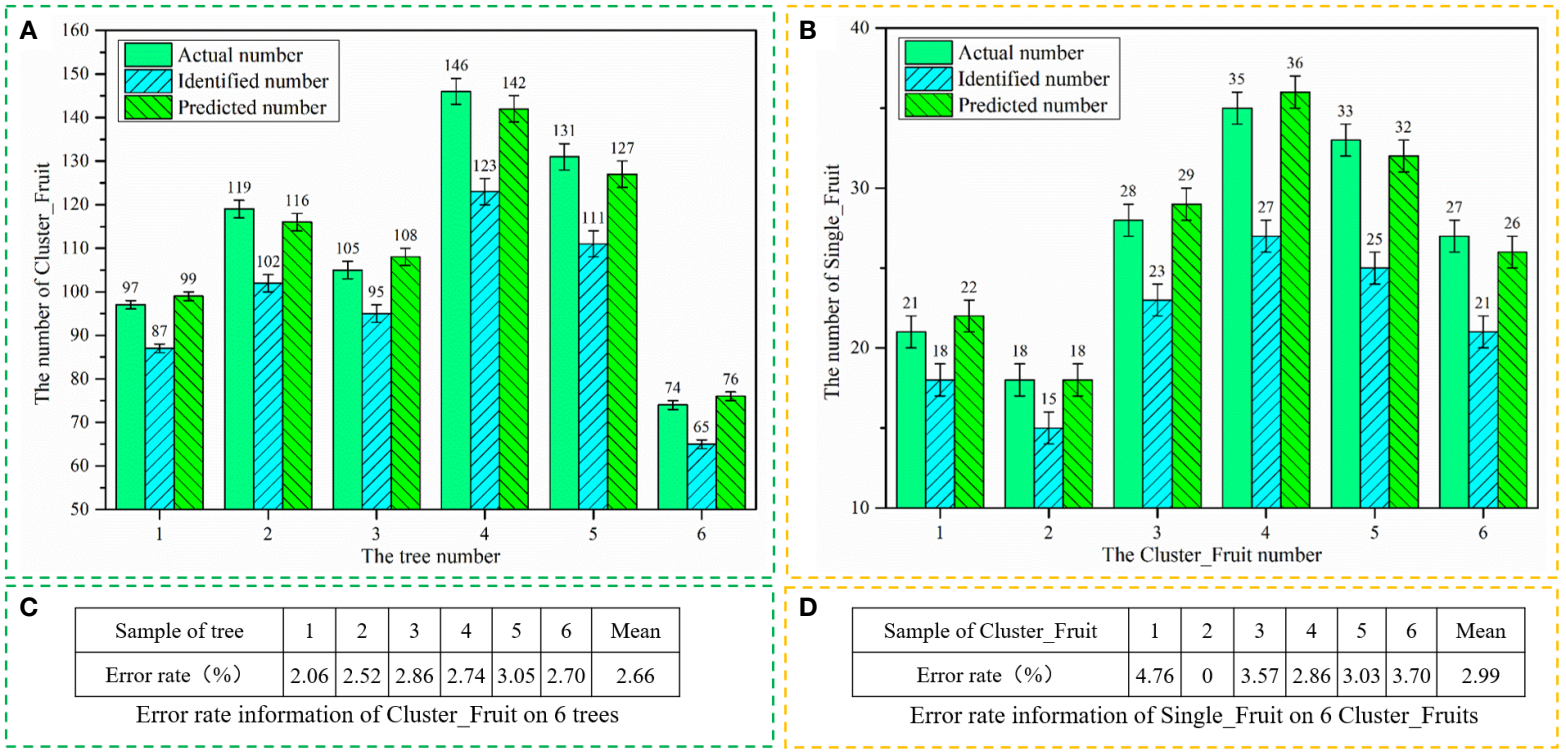
Statistical information of Cluster_Fruits and Single_Fruits. **(A)** The actual numbers, identified numbers and predicted numbers of Cluster_Fruits on six longan trees, **(B)** The actual numbers, identified numbers and predicted numbers of Single_Fruits on six Cluster_Fruits, **(C)** Error rate information of Cluster_Fruit on six trees, **(D)** Error rate information of Single_Fruit on six Cluster_Fruits.

The actual numbers, identified numbers and predicted numbers of Cluster_Fruits on six longan trees and Single_Fruits on 6 Cluster_Fruits are counted in **Figures 14A, B**, respectively, and their error rate data are counted in **Figures 14C, D**. It can be seen from the data in **Figures 14A, B** that the number of Cluster_Fruits identified by the CF-YD model and the number of Single_Fruits identified by the SF-YD model are corrected by the fitting equation obtained in Section 4.4, and the predicted numbers are very close to the actual numbers. According to the data in **Figure 14C**, the average error rate of Cluster_Fruit of 6 longan trees is 2.66%. According to the data in **Figure 14D**, the average error rate for the Single_Fruits of 6 Cluster_Fruits is 2.99%. It can be seen from the data in **Figures 14C, D** that the prediction error rates of Cluster_Fruits and Single_Fruits are below 5%.

According to the statistical results of Section 4.3, Section 4.4 and this section, there are errors between the real value and the identified value, which are mainly caused by two reasons. ① The two models have certain accuracy levels when detecting targets. ② The real value of Cluster_Fruits and Single_Fruits are obtained by manual statistics, which is a multiangle and full-range process. However, the UAV collects video images of the fruit tree canopy from the front angle and can only obtain Cluster_Fruit and Single_Fruit images outside the tree canopy.

According to the statistics of horticulture experts, the average fruit weight of the Chuliang longan variety is 13 g and that of the Shixia longan variety is 8 g. After using the method proposed in this paper to obtain the number of Cluster_Fruits on a single fruit tree and the number of Single_Fruits on each Cluster_Fruit, the yield data of a single longan tree can be obtained by using the yield estimation strategy for a single fruit tree in Section 3.4.

## Conclusion

In a complex longan orchard, fruit grow in clusters, and the shapes of Cluster_Fruits vary widely. It is difficult to estimate the yield of a single fruit tree simply by counting the number of Cluster_Fruits. Although the shapes Single_Fruits are relatively consistent, their shapes are small, so it is difficult to accurately count the number Single_Fruits directly by image analysis. Therefore, the yield estimation strategy based on UAV images proposed in this paper is of great significance and can improve the accuracy and efficiency of the yield statistics obtained for each fruit tree.

In this study, a method based on UAV images and computer vision technology is proposed to estimate the yield of a single longan fruit tree. First, a UAV is used to collect video images of the fruit tree canopy, and after preprocessing the images, two datasets are constructed, and the targets of the datasets are manually marked. Then, the CF-YD and SF-YD models are constructed to identify Cluster_Fruits and Single_Fruits, respectively, which realizes the task of automatically identifying the number of targets directly from each image. Finally, to further predict the yield of a single longan fruit tree accurately, two models for estimating the numbers of Cluster_Fruits and Single_Fruits are proposed, and two fitting equations are established for determining the actual number and predicted number of Cluster_Fruits on a single fruit tree and the number of Single_Fruits on a single Cluster_Fruit, and the models are tested and verified in real orchards. This study can quickly and accurately estimate the yield of a single fruit tree, which can not only provide guidance for the production management and market pricing of longan orchards but also improve the efficiency of deploying harvesting robots and transportation robots, which is conducive to maximizing the economic benefits of orchards. The research in this paper can apply UAV image migration to the harvests of clustered fruits such as grapes and Cerasus pseudocerasus and promote the development of smart agriculture and unmanned farms.

Since most longan orchards are currently unstructured, this work still has some limitations, and the target detection and tracking abilities of the proposed method need to be further improved. In this study, the UAV mainly collects canopy images of longan fruit trees from the perspective of elevation but cannot obtain all-around images inside the canopies of fruit trees. Therefore, there is an error between the collected data and the real values. In future research, we will first consider the use of UAV to automatically plan flight routes in order to obtain orchard canopy images more easily. Secondly, an image analysis processor will be built on the UAV to calculate the output of fruit trees in real time. Finally, the result data of artificial statistics will continue to be added to further improve the accuracy of the fitting equation prediction quantity. In addition, the research objects will be expanded to more longan varieties in the future. In future work, we will continue to optimize the details of the solution to promote the development of smart agriculture.

## Data availability statement

The raw data supporting the conclusions of this article will be made available by the authors, without undue reservation.

## Author contributions

DL and XS conceived the study and wrote the paper. YJ, ZY, PL, YC, HZ, ZZ, and KW participated in the experiment and analyzed the experimental data. JL and LS supervised the manuscript and made valuable inputs. All authors contributed to the article and approved the submitted version.

## References

[B1] Alpaydin E. (2016). Neural networks and deep learning. machine learning: The new AI. MIT Press. Cambridge, Massachusetts, USA. https://ieeexplore.ieee.org/document/7845182

[B2] Anagnostis A. Tagarakis A. C. Asiminari G. Papageorgiou E. Kateris D. Moshou D.. (2021). A deep learning approach for anthracnose infected trees classification in walnut orchards. Comput. Electron. Agric. 182. doi: 10.1016/j.compag.2021.105998

[B3] Bewley A. Ge Z. Ott L. Ramos F. Upcroft B. (2016). Simple Online and Realtime Tracking. 2016 IEEE International Conference on Image Processing (ICIP), Phoenix, AZ, USA, pp. 3464–3468. doi: 10.1109/ICIP.2016.7533003

[B4] Bochkovskiy A. Wang C. Y. Liao H. (2020). YOLOv4: Optimal speed and accuracy of object detection. doi: 10.48550/arXiv.2004.10934

[B5] da Silva C. B. Bianchini V. D. M. de Medeiros A. D. de Moraes M. H. D. Marassi A. G. Tannus A. (2021). A novel approach for jatropha curcas seed health analysis based on multispectral and resonance imaging techniques. Ind. Crops Prod. 161. doi: 10.1016/j.indcrop.2020.113186

[B6] de Medeiros A. D. Bernardes R. C. da Silva L. J. de Freitas B. A. L. Dias D. C. F. D. da Silva C. B. (2021). Deep learning-based approach using X-ray images for classifying crambe abyssinica seed quality. Ind. Crops Prod. 164. doi: 10.1016/j.indcrop.2021.113378

[B7] Ding X. Zhang X. Ma N. Han J. Ding G. Sun J. (2021). RepVGG: Making VGG-style ConvNets Great Again. 2021 IEEE/CVF Conference on Computer Vision and Pattern Recognition (CVPR), Nashville, TN, USA, pp. 13728–13737. doi: 10.1109/CVPR46437.2021.01352.

[B8] Feng A. J. Zhou J. F. Vories E. Sudduth K. A. (2020a). Evaluation of cotton emergence using UAV-based imagery and deep learning. Comput. Electron. Agric. 177. doi: 10.1016/j.compag.2020.105711

[B9] Feng A. J. Zhou J. F. Vories E. D. Sudduth K. A. Zhang M. N. (2020b). Yield estimation in cotton using UAV-based multi-sensor imagery. Biosyst. Eng. 193, 101–114. doi: 10.1016/j.biosystemseng.2020.02.014

[B10] Flores P. Zhang Z. Igathinathane C. Jithin M. Naik D. Stenger J.. (2021). Distinguishing seedling volunteer corn from soybean through greenhouse color, color-infrared, and fused images using machine and deep learning. Ind. Crops Prod. 161. doi: 10.1016/j.indcrop.2020.113223

[B11] Gao F. F. Fu L. S. Zhang X. Majeed Y. Li R. Karkee M.. (2020). Multi-class fruit-on-plant detection for apple in SNAP system using faster r-CNN. Comput. Electron. Agric. 176. doi: 10.1016/j.compag.2020.105634

[B12] Ge Z. Liu S. Wang F. Li Z. Sun J. (2021). YOLOX: Exceeding YOLO series in 2021. doi: 10.48550/arXiv.2107.08430

[B13] Girshick R. (2015b). Fast R-CNN. 2015 IEEE International Conference on Computer Vision (ICCV), Santiago, Chile, pp. 1440–1448. doi: 10.1109/iccv.2015.169

[B14] Girshick R. Donahue J. Darrell T. Malik J. (2015a). Region-based convolutional networks for accurate object detection and segmentation. IEEE Trans. Pattern Anal. Mach. Intell. 38 (1), 142–158. doi: 10.1109/tpami.2015.2437384 26656583

[B15] He L. Fang W. Zhao G. Wu Z. Fu L. Li R.. (2022). Fruit yield prediction and estimation in orchards: A state-of-the-art comprehensive review for both direct and indirect methods. Comput. Electron. Agric. 195. doi: 10.1016/j.compag.2022.106812

[B16] He Z. L. Xiong J. T. Chen S. M. Li Z. X. Chen S. F. Zhong Z.. (2020). A method of green citrus detection based on a deep bounding box regression forest. Biosyst. Eng. 193, 206–215. doi: 10.1016/j.biosystemseng.2020.03.001

[B17] He K. M. Zhang X. Y. Ren S. Q. Sun J. (2015). Spatial pyramid pooling in deep convolutional networks for visual recognition. IEEE Trans. Pattern Anal. Mach. Intell. 37 (9), 1904–1916. doi: 10.1109/tpami.2015.2389824 26353135

[B18] Jaisin C. Pathaveerat S. Terdwongworakul A. (2013). Determining the size and location of longans in bunches by image processing technique. Maejo Int. J. Sci. Technol. 7 (3), 444–455. doi: 10.14456/mijst.2013.37

[B19] Jiang T. Cheng J. (2019). Target Recognition Based on CNN with LeakyReLU and PReLU Activation Functions. 2019 International Conference on Sensing, Diagnostics, Prognostics, and Control (SDPC), Beijing, China, pp. 718–722. doi: 10.1109/SDPC.2019.00136.

[B20] Koirala A. Walsh K. B. Wang Z. L. McCarthy C. (2019). Deep learning - method overview and review of use for fruit detection and yield estimation. Comput. Electron. Agric. 162, 219–234. doi: 10.1016/j.compag.2019.04.017

[B21] Li D. H. Sun X. X. Elkhouchlaa H. Jia Y. H. Yao Z. W. Lin P. Y.. (2021). Fast detection and location of longan fruits using UAV images. Comput. Electron. Agric. 190. doi: 10.1016/j.compag.2021.106465

[B22] Li D. H. Sun X. X. Lv S. P. Elkhouchlaa H. Jia Y. H. Yao Z. W.. (2022). A novel approach for 3D localization of branch picking points based on deep learning applied to fruit picking UAVs. Comput. Electron. Agric. 199. doi: 10.1016/j.compag.2022.107191

[B23] Liang C. X. Xiong J. T. Zheng Z. H. Zhong Z. Li Z. H. Chen S. M.. (2020). A visual detection method for nighttime litchi fruits and fruiting stems. Comput. Electron. Agric. 169. doi: 10.1016/j.compag.2019.105192

[B24] Lin T. Y. Dollar P. Girshick R. He K. Hariharan B. Belongie S. (2017). Feature pyramid networks for object detection. 2017 IEEE Conference on Computer Vision and Pattern Recognition (CVPR), Honolulu, HI, USA, pp. 936–944. doi: 10.1109/CVPR.2017.106

[B25] Lin G. Tang Y. Zou X. Cheng J. Xiong J. (2020). Fruit detection in natural environment using partial shape matching and probabilistic hough transform. Precis. Agric. 21 (1), 160–177. doi: 10.1007/s11119-019-09662-w

[B26] Liu S. Qi L. Qin H. Shi J. Jia J. (2018). Path aggregation network for instance segmentation. 2018 IEEE/CVF Conference on Computer Vision and Pattern Recognition, Salt Lake City, UT, USA, pp. 8759–8768. doi: 10.1109/CVPR.2018.00913

[B27] Ma L. Liu Y. Zhang X. L. Ye Y. X. Yin G. F. Johnson B. A. (2019). Deep learning in remote sensing applications: A meta-analysis and review. Isprs J. Photogramm. Remote Sens. 152, 166–177. doi: 10.1016/j.isprsjprs.2019.04.015

[B28] Marani R. Milella A. Petitti A. Reina G. (2021). Deep neural networks for grape bunch segmentation in natural images from a consumer-grade camera. Precis. Agric. 22 (2), 387–413. doi: 10.1007/s11119-020-09736-0

[B29] Norouzzadeh M. S. Nguyen A. Kosmala M. Swanson A. Palmer M. S. Packer C.. (2018). Automatically identifying, counting, and describing wild animals in camera-trap images with deep learning. Proc. Natl. Acad. Sci. United States America 115 (25), E5716–E5725. doi: 10.1073/pnas.1719367115 PMC6016780 29871948

[B30] Paoletti M. E. Haut J. M. Plaza J. Plaza A. (2019). Deep learning classifiers for hyperspectral imaging: A review. Isprs J. Photogramm. Remote Sens. 158, 279–317. doi: 10.1016/j.isprsjprs.2019.09.006

[B31] Pham V. T. Herrero M. Hormaza J. I. (2015). Phenological growth stages of longan (Dimocarpus longan) according to the BBCH scale. Sci. Hortic. 189, 201–207. doi: 10.1016/j.scienta.2015.03.036

[B32] Redmon J. Divvala S. Girshick R. Farhadi A. (2016). You only look once: Unified, real-time object detection. 2016 IEEE Conference on Computer Vision and Pattern Recognition (CVPR), Las Vegas, NV, USA, pp. 779–788. doi: 10.1109/CVPR.2016.91

[B33] Redmon J. Farhadi A. (2018). YOLOv3: An incremental improvement. arXiv e-prints. doi: 10.48550/arXiv.1804.02767

[B34] Ren S. Q. He K. M. Girshick R. Sun J. (2017). Faster r-CNN: Towards real-time object detection with region proposal networks. IEEE Trans. Pattern Anal. Mach. Intell. 39 (6), 1137–1149. doi: 10.1109/TPAMI.2016.2577031 27295650

[B35] Singh P. Verma A. Alex J. S. R. (2021). Disease and pest infection detection in coconut tree through deep learning techniques. Comput. Electron. Agric. 182. doi: 10.1016/j.compag.2021.105986

[B36] Sumesh K. C. Ninsawat S. Som-ard J. (2021). Integration of RGB-based vegetation index, crop surface model and object-based image analysis approach for sugarcane yield estimation using unmanned aerial vehicle. Comput. Electron. Agric. 180. doi: 10.1016/j.compag.2020.105903

[B37] Tang Y. C. Zhou H. Wang H. J. Zhang Y. Q. (2023). Fruit detection and positioning technology for a camellia oleifera c. Abel orchard based on improved YOLOv4-tiny model and binocular stereo vision. Expert Syst. Appl. 211, 118573. doi: 10.1016/j.eswa.2022.118573

[B38] Tetila E. C. Machado B. B. Menezes G. K. Oliveira A. D. Alvarez M. Amorim W. P.. (2020). Automatic recognition of soybean leaf diseases using UAV images and deep convolutional neural networks. IEEE Geosci. Remote Sens. Lett. 17 (5), 903–907. doi: 10.1109/LGRS.2019.2932385

[B39] Vanegas F. Bratanov D. Powell K. Weiss J. Gonzalez F. (2018). A novel methodology for improving plant pest surveillance in vineyards and crops using UAV-based hyperspectral and spatial data. Sensors 18 (1). doi: 10.3390/s18010260 PMC5795822 29342101

[B40] Wang C. Y. Bochkovskiy A. Liao H. (2021). “Scaled-YOLOv4: Scaling cross stage partial network,” in 2021 IEEE/CVF Conference on Computer Vision and Pattern Recognition.

[B41] Wang C. Y. Bochkovskiy A. Liao H. (2022). YOLOv7: Trainable bag-of-freebies sets new state-of-the-art for real-time object detectors. arXiv e-prints. doi: 10.48550/arXiv.2207.02696

[B42] Wang C. L. Luo T. H. Zhao L. J. Tang Y. C. Zou X. J. (2019). Window zooming–based localization algorithm of fruit and vegetable for harvesting robot. IEEE Access 7, 103639–103649. doi: 10.1109/access.2019.2925812

[B43] Wojke N. Bewley A. Paulus D. (2017). Simple online and realtime tracking with a deep association metric. 2017 IEEE International Conference on Image Processing (ICIP), Beijing, China, pp. 3645–3649. doi: 10.1109/ICIP.2017.8296962

[B44] Wu F. Y. Duan J. L. Ai P. Y. Chen Z. Y. Yang Z. Zou X. J. (2022). Rachis detection and three-dimensional localization of cut off point for vision-based banana robot. Comput. Electron. Agric. 198, 107079. doi: 10.1016/j.compag.2022.107079

[B45] Xiong J. T. He Z. L. Lin R. Liu Z. Bu R. B. Yang Z. G.. (2018). Visual positioning technology of picking robots for dynamic litchi clusters with disturbance. Comput. Electron. Agric. 151, 226–237. doi: 10.1016/j.compag.2018.06.007

[B46] Xiong J. T. Liu Z. Chen S. M. Liu B. L. Zheng Z. H. Zhong Z.. (2020). Visual detection of green mangoes by an unmanned aerial vehicle in orchards based on a deep learning method. Biosyst. Eng. 194, 261–272. doi: 10.1016/j.biosystemseng.2020.04.006

[B47] Zhong Z. Xiong J. T. Zheng Z. H. Liu B. L. Liao S. S. Huo Z. W.. (2021). A method for litchi picking points calculation in natural environment based on main fruit bearing branch detection. Comput. Electron. Agric. 189. doi: 10.1016/j.compag.2021.106398

[B48] Zhou Y. H. Tang Y. C. Zou X. J. Wu M. L. Tang W. Meng F.. (2022). Adaptive active positioning of camellia oleifera fruit picking points: Classical image processing and YOLOv7 fusion algorithm. Appl. Sciences-Basel 12 (24), 12959. doi: 10.3390/app122412959

[B49] Zhou J. Zhou J. F. Ye H. Ali M. L. Nguyen H. T. Chen P. Y. (2020). Classification of soybean leaf wilting due to drought stress using UAV-based imagery. Comput. Electron. Agric. 175. doi: 10.1016/j.compag.2020.105576

[B50] Zhuang J. J. Hou C. J. Tang Y. He Y. Guo Q. W. Zhong Z. Y.. (2019). Computer vision-based localisation of picking points for automatic litchi harvesting applications towards natural scenarios. Biosyst. Eng. 187, 1–20. doi: 10.1016/j.biosystemseng.2019.08.016

